# Society-Based Infection Prevention and Control Guidelines After COVID-19: A Delphi Study

**DOI:** 10.1155/jonm/8255757

**Published:** 2025-09-10

**Authors:** Loai Alfarajat, Marwa Al Barmawi, Ahmed Alnawafleh, Lourance Al Hadid

**Affiliations:** ^1^Nursing Department, Faculty of Nursing, Al-Balqa Applied University, Salt 19117, Jordan; ^2^Nursing Department, Faculty of Nursing, Al-Zaytoonah University of Jordan (ZUJ), Amman, Jordan; ^3^Adult Health Nursing Department, College of Nursing, Mutah University, Karak, Jordan

**Keywords:** community health, control, Delphi, infection, Jordan, prevention

## Abstract

**Background:** Current infection prevention and control guidelines, protocols, and practices for public, outside healthcare facilities are not adequate as evidenced by increasing rates of infections and outbreaks. This study proposed to formulate a consensus on the guidelines that would then govern future public health-related infection prevention and control practices.

**Methods:** A three-round Delphi technique was used to generate experts' consensus on the development and required modifications of the current IPC guidelines to address public safety. Eighteen infection prevention and control practitioners and experts were interviewed individually during the three rounds.

**Results:** Three themes identified during the first round: first, policies and regulations; second, curricula reforms and education for all levels (such as kindergartens, schoolers, and the public); and third, the content of the IPC public manual. Results from Rounds I, II, and III changed a few subcategories until experts agreed on the themes and the subthemes. It is required that one department be responsible for providing IPC and the needed training of employees and public individuals in nonhealthcare institutes.

**Conclusion:** Through a semistructured Delphi consensus process, this study formulated key guidelines for society-based IPC outside the healthcare settings. The resulting consensus emphasizes the need for standardized policies and regulations, comprehensive education across all societal levels, and a unified public IPC manual. Crucially, the findings mandate establishing a designated authority responsible for coordinating IPC implementation training and oversight across all nonhealthcare sectors to enhance public safety post-COVID-19.

## 1. Introduction

Infection prevention and control (IPC) guidelines refer to the policies and procedures implemented to prevent and control dissemination of infections or the spread of infection-causing microorganisms within all areas where humans are present, whether for work or to perform personal hobbies and activities [1]. The main aims of these guidelines include the reduction of infection rates through following strict lines of practice that are supported by evidence from scientific work, like clinical and laboratory studies [2].

Infection control in its formal form was established in the early 1950s in the United States, and only a small number of hospital-initiated infection control programs were established to minimize healthcare-associated infections (HAIs) [3]. These programs implemented infection surveillance by adopting basic epidemiological principles to elucidate risk factors for HAIs [4]. However, little knowledge exists to date about infection rates in areas other than healthcare facilities.

The interest in applying IPC guidelines in nonhealth settings, like schools and food-processing industrial sites, is essential to promote better preventive measures to preventable infections in the society. As the infection is defined as the transmission of pathogenic microorganisms, which successfully infest and colonize to show symptoms, this definition includes all microorganisms, such as viruses, bacteria, and fungi [5, 6]. Infection is not exclusively a healthcare-associated event. This infection can be transmitted from one person to another, through respiratory droplets, body fluids, direct exposure to nonhuman resources of the infectious agent, or even during childbirth from the mother to her born fetus [7, 8]. Therefore, it is imperative to address IPC in such places to promote this concept in the society, which could also reflect on individual practices in or outside the healthcare institutes and establish better health literacy among the nonhealthcare personnel [9–11].

Current practices in public commonplaces concerning nonhealthcare settings have become inadequate to stop the chain of infection. Therefore, this study revisited the current IPC guidelines within the COVID-19 outbreak context, to inform healthcare practitioners and society at large, for more responsive and comprehensive IPC guidelines in the COVID-19 aftermath, with elements of practice crucial for the development of more responsive and comprehensive IPC guidelines [12, 13].

The current outbreak of COVID-19 has raised questions about the adequacy of current IPC guidelines, protocols, and practices in public facilities, other than healthcare facilities [13]. The current adopted practice of physical distancing and the use of PPEs could be otherwise inadequate, as long-time practices and other ones could be hypothesized to stand as more practical and more convenient practices. The sought-out guidelines would represent better, easy-to-adopt practices that could then be infused in the public through teaching them to teachers in schools and universities, who would then transfer them to their students at different levels and based on the level of the students. The easy and slow infusion of these guidelines using age-appropriate instruments and methods that would be carried out convincingly and appropriately should ensure the production of more adherent persons as they understand reasonably why to adopt these practices and would achieve reliable outcomes that stand for long periods. The result could be a more IPC effectiveness in the community, less prevalence of cross-infections, and better adherence to guidelines assumed to bring good outcomes to health in simple and understandable language subsumed by the public and for all age groups.

There is limited research, particularly before and during the pandemic investigating the need for change in the IPC practices and approaches among the public, and the need to explore areas where a lack of clarity existed [12]. This study investigated, using a Delphi approach, the opinions of experts in the field to formulate a consensus on the guidelines that would then govern future public health-related IPC practices, thus enhancing public health using responsible and practical approaches [14].

### 1.1. Objectives of the Study

• Determine critical elements, especially for the IPC guidelines gaps that need to be addressed after COVID-19 to ensure public safety in nonhealthcare settings.
• Generate a consensus among a group of experts (i.e., experts in the field of IPC, researchers, and epidemiologists) on the elements of change in the society-based IPC guidelines and policies that should be changed in the COVID-19 aftermath.

## 2. Materials and Methods

A multiround Delphi technique was employed in this study to generate experts' consensus on the required modifications of the current IPC guidelines to address social safety. The Delphi technique has been used intensively to revise medical practice guidelines, nursing practice guidelines, and multidisciplinary guidelines. This study also used the Delphi study to generate an agreement among IPC experts and practitioners on IPC manual content that could be utilized to educate and train individuals from different nonhealthcare settings and age groups on proper practices. This study is a primary effort to decrease the prevalence of cross-infections and contamination in nonhealthcare institutes.

### 2.1. Design

Delphi, three-round, prospective design was used in this study. The Delphi survey method was used to forecast future scenarios and determine the range of opinions on the future of IPC guidelines [15]. This method is suitable due to the nature of the topic as an ever-lasting process of change.

The study comprised three rounds of interviews with the IPC experts:

a. The first round (i.e., idea generation) was addressed by all selected participants, who were asked to respond to general questions concerning the IPC guidelines for the public outside healthcare institutions. An interview guide was prepared by the authors, who agreed on the content, order, configuration, and flow of the questions and possible subdiscussions of the interview. The authors conducted a recruitment process for the data collectors and research assistants (the recruitment included advertisement, sorting applications, and applicants' interview). Furthermore, a workshop to train the data collectors and research assistants on the special mission was held in the Nursing College at Al-Balqa Applied University.
b. The second round came after the researchers agreed on the summary of the points, which were included as essential changes to the IPC guidelines. The collated ideas were arranged to represent how many times they were repeated by different experts. The summarized points were sent to the experts, who responded to the first round only. All experts responded to the study interview and showed interest in spending time in the interviews. They all agreed to have their names mentioned at any time during the study and after it is finished. They were all interested in participating in the second round after themes and items of the study questionnaire and the IPC manual were prepared. In this round, experts were asked to confirm, refine, or add to the agreed sum of points to qualify them for the third and final round.
c. After receiving the suggested changes from experts in Round II, the researcher conducted a thematic analysis and extracted the main themes and the subthemes from the suggestions. The resulting items or areas (of change on the IPC guidelines) were summarized and sent to the experts for final review and agreement. A consensus list that represented items of agreement among a group of experts composing the IPC guidelines was prepared. These items should be considered to improve health practices and outcomes regarding cross-infections and contamination.

### 2.2. The Experts

In this study, we recruited a group of experts, who were selected for their expertise in IPC and epidemiology (public health). The number of experts was 18, a suitable number for this type of study [16].

#### 2.2.1. Inclusion Criteria

a. Infection control researchers
b. Infection control practitioners (prevention)
c. Public health researchers
d. Public health practitioners

The specific criteria for each expert included in this study are as follows:

#### 2.2.2. The Researchers

a. A Ph.D. prepared or equivalent.
b. Leading research teams in infection control projects.
c. Published original studies on infection control topics.
d. Published work in English and indexed journals.

#### 2.2.3. Practitioners

At least a Master's prepared practitioner, or 10 years or more of experience in infection control.

a. Clinical experience in infection control practice (of at least 5 years for master's degree holders, 10 years for BSc).
b. Competent in reading and writing English.
c. Public health researchers or public health practitioners

### 2.3. The Domains (Themes) Addressed by the Experts

This document was developed to be used by infection control staff, epidemiologists, healthcare administrators, and public health administrators responsible for developing, implementing, and evaluating IPC for the public in different walks of life. The themes of the interview were addressed in the following questions, which were submitted to the experts during the interview of Round I:

• Do we focus on public awareness of information on the basic principles of hand hygiene, barrier precautions, safe work practices, and isolation practices (in certain situations)?
• Which aspects of the public health IPC guidelines are adequate to prevent infection measures? Please state as many as apply.

What aspects of the public IPC practice/guidelines require revision and what do you suggest improving them:

• Political measures and public places' infection control measures, e.g., provision of fiscal and human resources for maintaining IPC and occupational health programs, which are implemented as a response to emerging needs, preparedness for possible outbreaks, the presence of a disaster plan, and employee preparedness to be part of the implementation of any disaster/outbreak plan.
• Adequate organizational characteristics (e.g., staffing, establishment of a safety culture, distancing) influence employee/staff/person adherence to recommended IPCs, organizational safety culture or climate, and administrative involvement in the development and support of IPC programs.
• Education awareness of personal safety measures—in times of the outbreak and the flat times—Influence of group norms regarding acceptable safety practices; the organization's socialization process for new personnel, the use of automated sinks, measures that estimate adherence to hand washing especially after using the bathroom before and after having meals; and education about the important role of all standard and other types of infection prevention precautions in protecting members of the society.
• The use of antimicrobial, disinfectants, sanitizers, and other surface cleansing solutions in the workplace, living places, and other public places. Include infection control as a compulsory requirement in all medical and health allied faculties to increase the student's awareness as they are in high and continuous exposure to different types of infections in their clinical placement settings. Moreover, include a separate chapter that covers the infection control precautions and guidelines in all medical or allied health alternative courses that are offered to students from different nonhealth faculties.
• Include these precautions and guidelines in the school curriculum starting from kindergarten 1 (KG1) until year 12 based on the student's academic and intellectual levels.
• Methods, frequency, and mode of surveillance in different workplaces, and other places delivering services to the public, e.g., oversight of employee health services related to IPC, assessment of risk, and administration of recommended treatment following (suspected) exposure to hazardous/infectious agents.
• IPC precautions, including availability of PPEs, and other special safety equipment, respirator—N95 or higher filtration—to prevent inhalation of infectious particles, goggles, masks, etc.
• Environmental measures and decolonization, e.g., detailed recommendations for disinfection and sterilization of surfaces and medical equipment that have been in contact with prion-containing tissue or high-risk body fluids, and for cleaning of blood and body substance spills, management of solid waste, and use of detergents used sufficiently to decontaminate eating utensils and other sensitive/personal equipment, including public bathrooms.

### 2.4. The Rounds

#### 2.4.1. Round I

Round I comprised searching, contacting, and arranging field visits and interviews with the experts. The interviews were audio-recorded after having the approval of each expert. Open-ended questions were used to address elements of current IPC practice (explained above). During each meeting, the interview method, which can be completed by the expert without needing to make any effort, was employed. This method provides flexibility and time for the experts and the researchers. The experts have the right to freely answer the questions. This method shortened the time of the study based on the plan. This round took approximately 4 months, and the average time for the interviews was 37 min.

#### 2.4.2. Round II

The Round II interview guide was constructed from the data collated from Round I, plus a consensus list (a tick box style). Pattern recognition, categorization, and thematic analysis were employed by the principal researcher and were used to construct the Round II interview guide and the consensus list. This was used by using manual thematic analysis to achieve all sensitive meaning based on the cultural use of terms or reference, leading to better precision in the results [17]. The interview guide helped the interviewers to drive the experts for their opinions, words, additions, changes, or removal of items they view as unnecessary and may not serve the purpose of the study. At least one visit or a virtual meeting via a video conference was made for each expert to revise and ensure a proper understanding of the concepts of the consensus and to document new additions. These additions were again documented, patterns were refined, and categories were identified using qualitative analysis software. This step took 10 weeks.

#### 2.4.3. Round III

Upon completing Round II, consensus interview analyses, the researchers prepared the items as emphasized by the experts and listed them in a final checklist supplementary to the final interview guide. The purpose of Round III was to invite experts to consider their scores, considering the overall responses from the group, and to decide which items they would consider necessary to keep and which to remove or modify. The checklist, which constituted the agreed items, was sent for the experts to say their final words. The feedback percentages provided scores for each item, which made it easier for the experts to decide. After receiving the final checklist, the researchers checked the items for any changes so that any re-analysis could be carried out. Responses from this round revealed the consensus among all experts on the items refined from the previous round. Percentages, medians, interquartile ranges, means, and standard deviations were calculated for the resulting items/themes and subthemes.

Results are presented in terms of numbers from Round I to Round III. In addition, items of debate among the experts will be listed. Finally, agreements, the level of agreements, and the debatable items are listed to show the process of refining items that the experts agreed on. All experts' names are acknowledged in the final version of the study report as they approved to mention their names. External experts were provided as experts to ratify the consensus and ensure the comprehensive applicability of the agreed themes during Round III.

### 2.5. Pilot Testing

Three experts were asked to complete the study questionnaire from Round I. This step was necessary to determine the timeframe, readability, and relevance of the questions. In addition, this step is necessary to erase ambiguous, repetitive, or inaccurate items. Results from this step were excluded from the final study report.

### 2.6. Credibility and Trustworthiness

The research assistants used open-ended questions during the interview. The research assistants started the interview by asking the panelists to describe their current experience in general. This opening question was important for both the research assistants and the panelists to establish a dialog in a friendly manner and helped in responding to the other questions smoothly and comfortably. Each panelist was encouraged to share feelings, thoughts, and beliefs about their experience with very limited interruptions from the research assistant. The average time of the interview was 37 min, with a range of 29 and 48 min, excluding the preparation and testing time for the interview. The interview notes were transcribed verbatim within 24 h based on the audio records. The narratives of the interview were provided to the woman to approve, modify, or correct any information within 48 h from the interview. The researchers believed that data saturation was reached after achieving the current total of interviews. The process of analysis included reading and re-reading the data and themes by the researchers of the study. The researchers examined the themes, performed a cross-checking, and assessed whether these reflected the topic of the study. Analyst triangulation has been followed, whereas the researchers reviewed the findings.

### 2.7. Data Analysis

We analyzed data using Van Manen's hermeneutic approach using thematic analysis [18]. Manen's approach provided us with a way to understand how individuals experienced the surrounding world. An individual rationalistic confirmation, which determines decisions concerning, for instance, life, health, or family, operates on the assumption that human life is made intelligible and accessible to human reason in both broad and definitive manner. Therefore, the researchers assumed that panelists' decisions have been influenced by their intelligence and by their social factors, all of which this study tried to explore. This approach comprises four steps of data analysis aiming at crafting the texts to develop the patterns of meanings to the themes. These four steps are as follows: uncovering thematic proportions and aspects; isolating statements that reflect each theme; composing linguistic transformations; and gleaning descriptions that represent the themes [19]. It has been stipulated that the four-step analysis would allow us to understand the meaning of the experience of the women as they lived in it and the knowledge would provide services or strategies to help the panelists more effectively. The researchers transcribed interview records and read and re-read them twice; transcription was performed by researcher assistants who conducted the interview in Arabic and translated the version allowing immersion in the data [20]. The researchers highlighted significantly broader patterns of phrases as possible patterns and themes. Similar meanings were grouped as categories and were clustered to create patterns. The researchers then grouped these patterns as themes and labeled them. The researchers made a critical comparison of the emerging themes against the categories to ensure that no other patterns could be identified from the dataset. After thorough discussions among the researchers, which included comments and suggestions by the panelists, a consensus was reached to include only the identified themes. During the second and the third rounds, the research assistants met with the panelists with specific items where consensus was sought among them. The third round was specifically run to match agreed-on items, refine the findings, and determine the exact meanings of each item for the final report of results.

## 3. Results

Data were collected between October 2022 and December 2023. The first round was conducted by setting up in-person face-to-face meeting, with each member of the experts. They all agreed to give their time to participate in the study. During the analysis of Round I interviews, three themes were identified with their subcategories (Table 1). Results from Round I, Round II, and Round III changed a few subcategories until experts agreed on the themes and the subthemes under these themes. The following reports each of those three themes and the corresponding subthemes.

### 3.1. Policies and Regulations

The first theme that emerged from the data was Policies and Regulations. Furthermore, all its subthemes were identified during this phase with a few modifications during the second round (Table 2).

### 3.2. Curricula Reforms and Education for All Levels

The second theme identified during the first-round interviews was curriculum reforms and education for all levels. All its subthemes also identified during this round, while no modifications were requested from any participant during the consequent rounds (Table 3).

### 3.3. Content of IPC Public Manual

All the participants emphasized the importance of developing an IPC manual for use by the public. Based on the interviews of both the epidemiologist and the practitioners, we identified the contents of this manual. The proposed table of contents derived from the first-round interviews analysis was approved by all participants during Round II and IV (Table 4).

Most of the items were not changed between Rounds II and III. Only a few experts reported worries about the independence of the department responsible for the IPC in different nonhealthcare settings. Experts agreed on the importance of having a single department that controls, decides on the measures, and takes the necessary steps to ensure nonhealth organizations' adherence to and implementation of IPC policies and guidelines. According to the experts, it is necessary to have an independent department with qualified personnel, who can do the steps they find essential to ensure public safety through the adoption of law-supported IPC guidelines. They requested that this department receives financial support from the government to run independently without interference from other governmental or private personnel. Similarly, some experts added that each industry receives the training, follows, and evaluates IPC guidelines based on recommendations from experts in that field so that appropriate items are included in this process. They also supported the need to have a frequent revision of the IPC guidelines to emphasize up-to-date and evidence-based practices that improve public well-being. An additional important point in this study is that the experts emphasized that each industry receives its training needs based on an actual need assessment keeping in mind the language and the content appropriate for the recipients, e.g., food industry workers, school students, and teachers.

The content of the manual represented common themes usually required by the IPC experts to train personnel in any industry. Despite being similar in the headings, the content of these chapters should be modified based on the recipients' age, level of maturity, and environmental components that determine the IPC content. Therefore, the proposed content of the IPC manual can be modified based on the needs and capacity of the recipients.

## 4. Discussion

The study aimed to reach a consensus among a group of experts, who are experts in different fields and deal with IPC in their different nonhealthcare settings. This study employed in-depth interviews, over three rounds with 18 experts from different nonhealthcare settings who have an expertise and interest in employing IPC guidelines and control measures to minimize the prevalence of cross-infections among individuals who do not belong to healthcare institutes in Jordan. This study provides evidence that indicates how important IPC employment is in different nonhealthcare institutes exhibited by the emphasis of all involved in this study on the importance of having common policies and guidelines governing IPC practices in nonhealth institutes. It also emphasizes that IPC should be observed, assessed, implemented, and evaluated by a single department with adequate legal power and personnel to enforce the implementation of the laws concerning IPC guidelines. In addition, this study shows that there is a need to develop a manual that addresses IPC training content for each of the industries involving human health, such as food-processing industries, and kindergartens. These findings can be applied in Jordan and other countries, which are similar or have comparable situations where nonhealthcare institutes' IPC measures are left unobserved and under control. These institutes, which represent different industries, have been left to struggle with very few or unclear IPC measures that belong merely to the improvisation of the governmental agency that is responsible for giving the license needed to keep it open.

In this study, the experts emphasized the need to have a single department that runs, regulates, develops, observes, revises, evaluates, and ensures that the regulations govern all issues concerning IPC guidelines and policies in nonhealthcare institutes. As emphasized in the literature, nonhealthcare employees and workers were left without specific IPC guidelines in many countries and industries; this also includes Jordanian institutes that provide services to individuals and could involve issues concerning human well-being and contamination that leads to cross-infections [21]. The discretionary use of IPC guidelines has always been a concern to many experts in the IPC field from different nonhealthcare settings. Therefore, these guidelines should be enforced to ensure the improvement of public health and minimize the prevalence of cross-contamination all over different industries, including long-term care facilities [21]. Whereas IPC in healthcare institutes is still a major challenge to many experts, it could be even more challenging in other industries. Therefore, trained personnel are needed to take over the responsibility of observing, evaluating, and ensuring the implementation of these guidelines all over these different nonhealthcare settings, especially those that influence many individuals [22]. Risk mitigation is essential in many industries, including contamination and cross-infection prevention. As emphasized in the literature, gaps in national IPC activities have a significant impact on each country's ability to meet the International Health Regulations, corresponding well-established scheme to disease outbreaks, and combat any endemics or pandemics, like COVID-19 [23]. Therefore, it is imperative to develop assessment tools and plan proper corrective measures that suit each industry. All of which this study emphasized in its proceedings.

This study provides recommendations to unify the assessment, evaluation, and follow-up of IPC issues in industries that involve human well-being. In the Jordanian case, it is required that one department be responsible for providing these services and the needed training of employees and individuals receiving services in nonhealthcare institutes to have one reference that provides IPC-related consultations to them, including the promotion of proper implementation of IPC guidelines as required by each industry [1]. As the WHO [24] report highlights the importance of integration of IPC interventions with water, sanitation, and hygiene (WASH) strategies in a broader context than the healthcare institute, similar efforts can be made toward nonhealthcare institutes to improve response to IPC issues. Hence, efforts aiming at maximizing the prevention of infection should also focus on populations dealing with settings of common interest to IPC, such as food-processing workers, kindergartens, and schools. Training employees, teachers, students, and children on IPC taking into consideration their level of knowledge and skills might help promote the IPC healthy practices among greater populations within the community [25]. It is hoped that improving IPC outcomes in nonhealthcare settings would reflect better outcomes in healthcare institutes, as well as influencing public knowledge and adherence to IPC good practices [26]. While current IPC programs emphasize evidence-based practices in healthcare institutes, similar emphasis should be placed also on nonhealthcare institutes. In that respect, community-based IPC manuals have been deployed in different countries [13], and similar manuals could not be found in others, like Jordan. Therefore, this study needed to address the content and components of the manual that could be proposed to train and enhance the skills of nonhealthcare personnel as well [27].

## 5. Conclusion

Through a semistructured Delphi consensus process, this study formulated key guidelines for society-based IPC outside the healthcare settings. The resulting consensus emphasizes the need for standardized policies and regulations, comprehensive education across all societal levels, and a unified public IPC manual. Crucially, the findings mandate establishing a designated authority responsible for coordinating IPC implementation training and oversight across all nonhealthcare sectors to enhance public safety post-COVID-19.

## Figures and Tables

**Table 1:** Themes identified from the study.

**No**	Theme title
**Theme I**	Policies and regulations
**Theme II**	Curriculum reforms and education for all levels (such as kindergartens, schoolers, and the public)
**Themes III**	Content of IPC public manual

**Table 2:** Subcategories identified for the theme—policies and regulations.

Theme I: policies and regulations	Phase II	Phase III
Unify infection prevention and control measures and procedures in nonhealthcare settings under one body, either a department or a committee that belongs to one of the ministries	Not changed	Not changed
Specify the juristic of this department/committee by the law emphasizing that it is the only one responsible for assessing, evaluating, and maintaining IPC practices adequately followed in nonhealth settings	Not changed	Not changed
Provide this body with the required staff, instruments, laboratories, and evaluation/assessment sheets to carry out its duties	Not changed	Not changed
Follow only one track of reporting. This includes canceling any overlaps that could occur once it is formed with other governmental departments or institutes	Not changed	Not changed
This body should be supported by the law with an executive arm that enforces the implementation of all IPC-required elements	Request from 3 panelists to change	Changed as requested
The financing of this body could be included by a contribution of all institutions on the annual relicensing expenses	Request from 3 panelists to change	Changed as requested
All reports coming out of this body should be treated as confidential and the public reporting of results should be carried out as per the governmental policies	Request from 5 panelists to change	Changed as requested

*The responsibilities of this body include*		
• Preparing an IPC manual with all its sections (each section addresses a particular industry or workplace)	Not changed	Not changed
• Preparing training material for the new staff in different settings	Not changed	Not changed
• Evaluating the readiness of each setting	Not changed	Not changed
• Conducting workshops to train trainees and trainers	Not changed	Not changed
• Running periodic inspection visits to ensure the proper implementation of IPC guidelines	Not changed	Not changed
• Preparing national reports including all results concerning outbreaks	Not changed	Not changed
• Participating in investigating any of these infection outbreaks	Not changed	Not changed

**Table 3:** Subcategories of Theme II—curriculum reforms and education for all levels.

Theme II: curriculum reforms and education for all levels (such as kindergartens, schoolers, and the public)		
Reforming the national curricula taught in: (adding age-appropriate content regarding IPC and HW to curricula and distributing this content across several years, covering topics that can be easily comprehended and have an impact on children's behaviors, beginning with HW easy habits in kindergartens, hand contamination, reaching to unneeded prescribed antibiotics (OCD), incomplete usage or left overuse of antibiotics, or MDR)	Not changed	Not changed
• Kindergartens	Not changed	Not changed
• Schools	Not changed	Not changed
• Colleges	Not changed	Not changed
• Universities	Not changed	Not changed
Guarantee that all employees in the public and private sectors are sufficiently informed about IPC practices	Not changed	Not changed
Staff members working in kindergartens should be educated and trained regarding the isolation and cleansing procedures of children, including the type and method by which isolation could be carried out for both individuals and groups	Not changed	Not changed
Create appealing IPC posters and flyers so that people would be drawn in and be able to comprehend the content with little effort	Not changed	Not changed
Child food containers and bottles are also taken to break the cycle of infection using the correct as well as effective procedures (evidence-based practice)	Not changed	Not changed
Specific recommendations for mothers and fathers should be changed, as well as IPC home practices. To integrate the process into the next generation's way of life, the parents should be considered as a very essential target	Not changed	Not changed
The following subjects should be covered in the curricula, but not exclusively:	Not changed	Not changed
• HW	Not changed	Not changed
• Personal hygiene	Not changed	Not changed
• Learning what IPC is	Not changed	Not changed
• Unneeded antibiotics that are not prescribed (OCD), or those that are prescribed but not used completely or in excess	Not changed	Not changed
• Food contamination and reservations	Not changed	Not changed
• Use of household cleaning supplies, tools, and detergents (including the use of antiseptics and sanitary solutions)	Not changed	Not changed
• Appropriate and age-appropriate instruction on how to use disinfectants	Not changed	Not changed
• PPEs	Not changed	Not changed
• Cleaning and at-home preservation of agricultural products	Not changed	Not changed
• Proper food handling and preserving	Not changed	Not changed
• How infection occurs and how to effectively break the cycle of infection utilizing straightforward techniques	Not changed	Not changed
• Isolation (justification and application)	Not changed	Not changed
• MDR hazards, with an appropriate explanation based on age	Not changed	Not changed
• How to deal with crowded areas properly	Not changed	Not changed
• Being aware of the unique requirements for various public spaces	Not changed	Not changed
• MDR and its relationship to OCD	Not changed	Not changed
• Proper waste disposal	Not changed	Not changed
• The importance of respiratory hygiene	Not changed	Not changed
Promoting awareness is a crucial necessity. Workshops should take into consideration that the public is not currently aware of the infection cycle	Not changed	Not changed

**Table 4:** Subthemes of Theme III: content of IPC public manual.

Round I theme subcategories and table of contents for the manual	Round II	Round III
Chapter 1: Introduction	Not changed	Not changed
Section 1: Background	Not changed	Not changed
Explain the purpose of the manual, which is to guide lay persons outside health settings on how to prevent and control infections in their everyday lives	Not changed	Not changed
Emphasize the importance of infection prevention and control in preventing the spread of infectious diseases	Not changed	Not changed
Provide an overview of the content of the manual	Not changed	Not changed
Section 2: Understanding infections	Not changed	Not changed
Define what an infection is and how it spreads	Not changed	Not changed
List common types of infections and their symptoms	Not changed	Not changed
Explain the importance of identifying and reporting infections	Not changed	Not changed
Chapter 2: Basic infection prevention measures	Not changed	Not changed
Describe basic infection prevention measures that can be taken in everyday life, such as washing hands, covering mouth and nose when coughing or sneezing, and staying home when sick	Not changed	Not changed
Guide proper hand hygiene techniques	Not changed	Not changed
Discuss the importance of avoiding close contact with others when sick	Not changed	Not changed
Hand hygiene	Not changed	Not changed
Personal hygiene	Not changed	Not changed
Respiratory management	Not changed	Not changed
Section 1: Hand hygiene	Not changed	Not changed
Why hand hygiene is important	Not changed	Not changed
When to perform hand hygiene	Not changed	Not changed
Proper handwashing technique	Not changed	Not changed
Use of hand sanitizer	Not changed	Not changed
Section 2: Personal hygiene	Not changed	Not changed
Explain the importance of good personal hygiene in preventing the spread of infections	Not changed	Not changed
Discuss the importance of hand hygiene	Not changed	Not changed
Explain how to properly wash hands	Not changed	Not changed
Discuss the importance of covering the mouth and nose when coughing or sneezing	Not changed	Not changed
Explain the proper disposal of tissues and other waste products	Not changed	Not changed
Section 3: Respiratory management	Not changed	Not changed
Importance of respiratory-related symptoms	Not changed	Not changed
How infections spread through coughs and sneezes	Not changed	Not changed
Proper cough and sneeze etiquette	Not changed	Not changed
Use of masks and face coverings	Not changed	Not changed
Chapter 3: Additional measures to prevent infection	Not changed	Not changed
Section 1: Social distancing	Not changed	Not changed
Explain the importance of social distancing	Not changed	Not changed
Discuss how to properly practice social distancing	Not changed	Not changed
Section 2: Personal protective equipment (PPE)	Not changed	Not changed
Define what personal protective equipment (PPE)	Not changed	Not changed
Importance of personal protective equipment	Not changed	Not changed
Types of personal protective equipment	Not changed	Not changed
Proper use of personal protective equipment	Not changed	Not changed
When to use personal protective equipment	Not changed	Not changed
Chapter 4: Specific situations	Not changed	Not changed
Section 1: Food safety	Not changed	Not changed
Importance of food safety	Not changed	Not changed
Common foodborne illnesses	Not changed	Not changed
Proper food handling techniques	Not changed	Not changed
Storage and preparation of food	Not changed	Not changed
Section 2: Travel precautions	Not changed	Not changed
Importance of travel precautions	Not changed	Not changed
How infections can spread during travel	Not changed	Not changed
Pre-travel preparations	Not changed	Not changed
During-travel precautions	Not changed	Not changed
Post-travel precautions	Not changed	Not changed
Section 3: Environmental hygiene	Not changed	Not changed
Discuss the importance of keeping the environment clean	Not changed	Not changed
Explain how to properly clean and disinfect surfaces	Not changed	Not changed
Discuss how to handle laundry	Not changed	Not changed
Explain the importance of proper ventilation	Not changed	Not changed
Section 4: Cleaning and disinfecting	Not changed	Not changed
Importance of cleaning and disinfection. Explain the difference between cleaning and disinfecting	Not changed	Not changed
How infections can spread through contaminated surfaces	Not changed	Not changed
Proper cleaning and disinfection techniques	Not changed	Not changed
Recommended cleaning products	Not changed	Not changed
Guide how to clean and disinfect surfaces and objects in the home	Not changed	Not changed
List common household disinfectants and how to use them safely	Not changed	Not changed
Chapter 5: Monitoring and reporting and additional resources	Not changed	Not changed
Section 1: Response to infection	Not changed	Not changed
Identifying symptoms	Not changed	Not changed
What to do if you are infected	Not changed	Not changed
What to do if someone else is infected	Not changed	Not changed
Home isolation and quarantine	Not changed	Not changed
Section 2: Dealing with a sick person	Not changed	Not changed
Discuss the proper ways to care for a sick person	Not changed	Not changed
Explain how to properly dispose of waste products	Not changed	Not changed
Discuss how to prevent transmission of the illness to others	Not changed	Not changed
Explain the importance of seeking medical attention	Not changed	Not changed
Section 3: Monitoring and reporting	Not changed	Not changed
Signs and symptoms of infections	Not changed	Not changed
Reporting procedures for infections	Not changed	Not changed
What to do if you suspect an infection	Not changed	Not changed
Links to reliable sources of information	Not changed	Not changed
Contact information for local health authorities	Not changed	Not changed
Glossary of terms	Not changed	Not changed
Provide a list of additional resources for further information on infection prevention and control	Not changed	Not changed
Chapter 6: Conclusion	Not changed	Not changed
Summarize the key points of the manual	Not changed	Not changed
Emphasize the importance of following infection prevention and control measures in everyday life to prevent the spread of infectious diseases	Not changed	Not changed
Encourage readers to share the information with their family, friends, and community	Not changed	Not changed
Podcast explaining easy-to-read material on IPC (supported by 2-D or 3-D visions)	Not changed	Not changed
By following the information provided in this manual, individuals can take important steps to prevent the spread of infections in their daily lives	Not changed	Not changed
Remember to always prioritize infection prevention and control for the safety of yourself and those around you	Not changed	Not changed

## Data Availability

The datasets generated during the current study are not publicly available due to privacy but are available from the corresponding author upon reasonable request.
